# Foetal testosterone and autistic traits in 18 to 24-month-old children

**DOI:** 10.1186/2040-2392-1-11

**Published:** 2010-07-12

**Authors:** Bonnie Auyeung, Kevin Taylor, Gerald Hackett, Simon Baron-Cohen

**Affiliations:** 1 Autism Research Centre, Department of Psychiatry, University of Cambridge, Douglas House, 18B Trumpington Rd, Cambridge, CB2 8AH, UK; 2 Department of Clinical Biochemistry, Addenbrooke's Hospital, Cambridge, CB2 2QQ, UK; 3 Department of Foetal Medicine, Rosie Maternity Hospital, Robinson Way, Cambridge, CB2 2SW, UK

## Abstract

**Background:**

Autism spectrum conditions have been characterised as an extreme presentation of certain male-typical psychological traits. In addition, several studies have established a link between prenatal exposure to testosterone and cognitive sex differences in later life, and one study found that foetal testosterone (FT) is positively correlated to autistic traits in 6 to 10 year-old children. In this study, we tested whether FT is positively correlated with autistic traits in toddlers aged 18-24 months.

**Methods:**

Levels of FT were analysed in amniotic fluid and compared with autistic traits, measured using the Quantitative Checklist for Autism in Toddlers (Q-CHAT) in 129 typically developing toddlers aged between 18 and 24 months (mean ± SD 19.25 ± 1.52 months).

**Results:**

Sex differences were observed in Q-CHAT scores, with boys scoring significantly higher (indicating more autistic traits) than girls. In addition, we confirmed a significant positive relationship between FT levels and autistic traits.

**Conclusions:**

The current findings in children between 18 and 24 months of age are consistent with observations in older children showing a positive association between elevated FT levels and autistic traits. Given that sex steroid-related gene variations are associated with autistic traits in adults, this new finding suggests that the brain basis of autistic traits may reflect individual differences in prenatal androgens and androgen-related genes. The consistency of findings in early childhood, later childhood and adulthood suggests that this is a robust association.

## Background

Autism, high-functioning autism, Asperger syndrome (AS) and pervasive developmental disorder not otherwise specified (PDD-NOS) are collectively referred to as autism spectrum conditions (ASC). Recent research has suggested that ASC represent the upper extreme of a collection of traits that are continuously distributed in the population [1,2]. This continuum view provides a shift away from the categorical diagnostic approach and towards a quantitative approach for measuring autistic traits.

The strong bias of ASC towards males is well established [3], with a clear male to female ratio, estimated at 4:1 for classic autism [4] and as high as 10.8:1 in individuals with AS [5]. The extreme male brain (EMB) theory of autism proposes that ASC are an exaggeration of certain male-typical traits [6,7]. This theory has been extended to explain both cognition and neuroanatomy in individuals with autism [8]. It has been suggested that prenatal exposure to testosterone may be a key biological mechanism for shaping sex differences in the brain, and may be involved in the biased sex ratio found in these conditions [8,9].

Although genetic sex is determined at conception, it is the gonadal hormones (that is, androgens, estrogens and progestins) that are responsible for differentiation of the male and female phenotypes in the developing human foetus [10]. Direct sampling of foetal serum or manipulation of foetal hormone levels would be very dangerous; hence, researchers have used indirect methods of measuring prenatal hormone exposure to study effects on later development.

One such indirect measure is the ratio between the length of the second and fourth digits (2D:4D) of the hand. This ratio has been found to be sexually dimorphic, being lower in males than in females. The 2D:4D ratio is thought to be fixed by week 14 of foetal life, and has been found to reflect foetal exposure to prenatal sex hormones in early gestation [11,12]. Results from studies of 2D:4D ratios as a proxy for FT levels show that children with ASC have more masculinised digit ratios compared with typically developing boys. These patterns have also been observed in the siblings and parents of children with ASC, indicating the possibility of a link between genetically based elevated FT levels and the development of ASC [13,14]. Other evidence of a genetic link to ASC was provided by a recent study, which showed that genes regulating sex steroids are associated with autistic traits, as measured by scores on the Autism Spectrum Quotient (AQ), in a typical adult sample [15]. A parallel study also showed that genes regulating sex steroids are associated with a diagnosis of AS in a case-control sample [15].

The medical condition of congenital adrenal hyperplasia (CAH) leads to abnormally high androgen levels, and has provided researchers with a 'natural experiment' in which to examine the effects of elevated androgen exposure. Girls with CAH have more autistic traits (measured using the adult AQ) than their unaffected sisters [16]. Given that this condition is usually treated after birth, this suggests that the higher AQ scores in such children reflect elevated prenatal androgen levels. However, these findings should be interpreted with caution, because CAH carries a number of related problems (and requires extensive treatment) which may affect the atypical cognitive profiles found in this population [17,18].

Some studies have also compared measurements of testosterone in umbilical cord (UC) blood with postnatal development. A recent study using UC blood testosterone measurements examined pragmatic language ability in girls followed up at 10 years of age. Results showed that the higher a girl's free testosterone level at birth, the higher the scores on a pragmatic language difficulties questionnaire [19]. However, levels of FT are typically at very low levels from about week 24 of gestation, and UC samples can contain blood from the mother as well as the foetus (and hormone levels may vary due to labour itself) [20], so UC blood testosterone does not allow testing of whether outcomes reflect FT *per se*.

Currently, the best method to examine the effect of FT is to sample via amniocentesis the amniotic fluid surrounding the foetus. An advantage of amniotic fluid samples is that amniocentesis is often performed for routine clinical purposes within a relatively narrow time period that coincides with the hypothesised critical period for human sexual differentiation between weeks 8 and 24 of gestation [21]. This is also more direct than the 2D: 4D method, as the hormones themselves can be assayed, rather than relying on a proxy for these.

Several studies have linked elevated levels of FT in the amniotic fluid with the masculinisation of certain behaviours, beginning shortly after birth. Elevated FT has been linked to reduced eye contact in infants, smaller vocabulary in toddlers, narrower interests at 4 years of age, less empathy at 4 and 8 years, and increased systemizing (the drive to analyse and construct systems) at 8 years [9,22-26]. In addition, FT levels have been found to be positively correlated with the number of autistic traits in older children (6-10 years old), using two independent dimensional measures of autistic traits (the AQ-Child version and the Childhood Autism Spectrum Test) [27].

The current study aimed to extend our understanding of the effect of prenatal hormones on the development of autistic traits, using a new measure for evaluating autistic traits in toddlers. The Quantitative Checklist for Autism in Toddlers (Q-CHAT) is a parent-report questionnaire that evaluates autistic traits in toddlers between 18 and 24 months old [28]. Scores on this measure have shown a near-normal distribution in a large general population sample (n = 779), suggesting that it is a useful measure of individual differences in autistic traits in toddlers [28]. In the present study, the relationship between measurements of FT and scores on the Q-CHAT were examined.

## Methods

This study was given ethical approval by the National Health Service Suffolk Research Ethics Committee. Written informed consent was obtained from general practitioners (GPs) and participating parents.

### Participants

Mothers were asked to participate in research at the time of having an amniocentesis (between 2004 and 2006). The medical records of approximately 700 amniocentesis patients were examined, and participants were excluded if: (1) there was a twin pregnancy; (2) amniocentesis revealed chromosomal abnormality; (3) the child suffered significant medical problems after birth or (4) records were incomplete. Women whose GP gave consent (n = 283) were contacted for participation. The final sample for this study included 129 participants (66 boys, 63 girls) with complete data.

### Outcome variable

The outcome variable was the Q-CHAT. This is a 25-item parent-report screening measure developed to identify toddlers at risk for development of ASC. This measure is a major revision of the Checklist for Autism in Toddlers (CHAT) which was a screening tool originally developed for use by health professionals for children aged between 18 and 24 months. Instead of using a binary (yes/no) format, the Q-CHAT uses a five-point scale of frequency of behaviour to allow a greater range of responses. The items of the Q-CHAT aim to measure areas of toddler development including joint attention (e.g., gaze-following, or using/following the index-finger pointing gesture), pretend play, language development, repetitive behaviours, and social communication. A total score is obtained with a minimum score of 0 and a maximum score of 100 [28]. The instrument was designed to have a broad range to best capture the dimensional nature of autistic traits in a general population.

### Predictor variable

The predictor variable was FT level. FT was measured from amniotic fluid by radioimmunoassay (at Department of Clinical Biochemistry, Addenbrooke's Hospital, Cambridge, UK) using a method that has been reported previously [24,27]. Amniotic fluid was extracted with diethyl ether. The ether was evaporated to dryness at room temperature, and the extracted material redissolved in an assay buffer. Testosterone was measured by a commercial assay ('Count-a-Coat'; Diagnostic Products Corp, Los Angeles, CA, USA), which uses an antibody to testosterone coated onto propylene tubes and a 125-I labelled testosterone analogue. FT level is expressed as nmol/L. The detection limit of the assay using the ether-extraction method is approximately 0.05 nmol/L. The coefficient of variation (CV) for between-batch imprecision is 19% at a concentration of 0.8 nmol/L, and 9.5% at a concentration of 7.3 nmol/L. CVs for within-batch imprecision are 15% at a concentration of 0.3 nmol/L, and 5.9% at a concentration of 2.5 nmol/L. This method measures total extractable testosterone.

### Control variables

#### Foetal estradiol (FE) levels

In rodents, conversion of FT to FE in the brain has been shown to be one mechanism for masculinisation of behaviour [29,30]. The role of estradiol in human development is unclear, and it has been suggested that testosterone directly influences sexual differentiation without being converted [31,32]. Measurements of FE therefore were included as a control variable to further examine whether there were any relationships with the other variables.

#### Gestational age at amniocentesis (in weeks)

Amniotic fluid samples were collected between weeks 11 and 21 of gestation (mean ± SD 16.92 ± 1.83). This timing coincides with the hypothesised critical period for human sexual differentiation (between approximately weeks 8 and 24 of gestation [9,21]).

#### Maternal age

Maternal age was included because women undergoing amniocentesis have a higher mean age than the general childbearing population.

#### Level of education obtained by parents

The mean maternal and paternal education level was computed. Parental education level was measured according to a five-point scale (1 = no formal qualifications, 2 = O level/General Certificate of Secondary Education (GCSE) or equivalent, 3 = A-level, Higher National Diploma (HND) or vocational qualification, 4 = university degree and 5 = postgraduate qualification).

#### Presence of older siblings

Older siblings have been found in previous research to have an effect on the social environment and influence child development [33]. This variable was defined as older brothers present in the home (or not) and older sisters present in the home (or not).

#### Child's age

The children included in the analyses were between 18 and 24 months of age (19.25 ± 1.52) at the time of Q-CHAT administration. Age was also included as a control variable.

## Results

### FT levels

Three male outliers in FT levels (individuals who scored 3 SDs above the mean) were observed. A winsorizing procedure was used [34], in which the extreme values were replaced by the highest observed level within 3SD from the mean (1.55 nmol/L). No outliers were found when FT levels were examined in girls. Winsorized FT levels showed no outliers and acceptable skewness statistics for both boys and girls, and were used in subsequent analyses. Independent samples *t*-tests showed significant sex differences in winsorized FT levels t_(110.93) _= 8.71, *P *< 0.001 (equal variances not assumed), with boys (0.80 ± 0.36) showing higher levels than girls (0.34 ± 0.23).

### Control variables

Examination of the univariate distributions of the control variables showed that levels of FE were positively skewed (skewness > 1) so a natural logarithmic transformation was carried out. This reduced the skewness to < 1, and transformed FE data were used in subsequent analyses. No sex differences were observed in transformed FE levels (*t*_(127) _= 0.22, *P *> 0.05). All other control variables showed acceptable distributions, with no outliers and no sex differences (all *P *> 0.05).

### Q-CHAT scores

**Figure 1:**
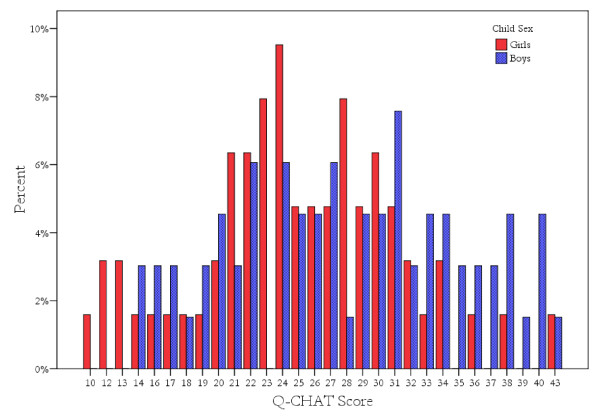
**Distribution of Quantitative Checklist for Autism in Toddlers (Q-CHAT) scores**.

Examination of the univariate distribution of Q-CHAT scores revealed that it was not significantly skewed (skewness < 1) for all cases together, or for boys and girls separately, thus the raw scores were used in further analyses. Figure 1 shows the distribution of Q-CHAT scores. Scores on the Q-CHAT showed significant sex differences (*t*_(127) _= 2.59, *P *= 0.01; equal variances assumed), with boys (28.09 ± 7.30) scoring higher than girls (24.94 ± 6.52) (see Table 1 for descriptive information and Q-CHAT score correlations with FT and the control variables). Effect sizes for sex differences were also computed using 'Cohen's d'. A 'd' value of 0.2 is considered a small effect size; a 'd' of 0.5, a medium effect size; and a 'd' > 0.8, a large effect size [35].

**Table 1:** Descriptive information and correlations with Quantitative Checklist for Autism in Toddlers (Q-CHAT) scores

	Combined group					Girls					Boys					Sex difference effect size (d)
	
Variable	n	Mean	SD	Range	*r*	n	Mean	SD	Range	*r*	n	Mean	SD	Range	*r*	
FT level^a^, nmol/L^c^	129	0.59	0.41	0.05 to 2.28	0.40**	63	0.34	0.27	0.05 to 1.12	0.31*	66	0.82	0.42	0.15 to 2.28	0.36*	1.36

FE level^a^, pmol/L	129	307.12	186.29	108 to 1260	0.01	63	309.67	181.98	126 to 1260	0.06	66	304.70	191.68	108 to 1220	-0.01	0.03

Gestational age	120	16.92	1.83	13 to 26	-0.13	60	17.01	1.62	15 to 22.3	0.06	60	16.83	2.03	13 to 26	-0.25	0.10

Child age	129	19.25	1.52	18 to 24	-0.15	63	19.40	1.50	18 to 23	0.08	66	19.11	1.53	18 to 24	-0.19	0.19

Maternal age	129	35.67	4.21	21 to 46	0.06	63	35.81	4.26	24 to 46	-0.01	66	35.53	4.18	21 to 45	0.14	0.07

Parental education	127	3.56	1.05	1 to 5	-0.02	61	3.58	1.05	1 to 5	0.12	66	3.54	1.06	1 to 5	-0.13	0.04

Q-CHAT Score^b^	129	26.55	7.08	10 to 43	-	63	24.94	6.52	10 to 43	-	66	28.09	7.30	14 to 43	-	0.46

^a^Indicates raw values.

^b^Sex difference is significant at the *P *< 0.05 level.

^c^Sex difference is significant at the *P *< 0.01 level.

*r *= correlation value between each variable and Q-CHAT scores.

* P < 0.05 level

** P < 0.01 level

**Figure 2:**
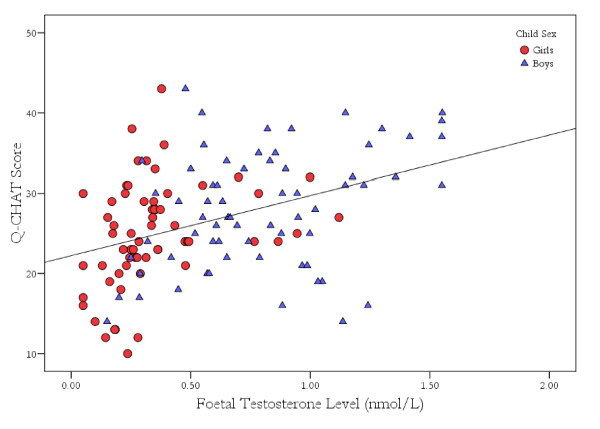
**Relationship between foetal testosterone (FT) level and Quantitative Checklist for Autism in Toddlers (Q-CHAT) scores**.

Data were examined using a hierarchical multiple regression analysis to determine potential predictors of Q-CHAT scores. In the first stage, any predictor variable showing a significant correlation with the outcome at the *P *< 0.20 was entered into the analysis [36]. The only variable meeting this criteria was gestational age (*r *= -0.13, *P *< 0.20), which was included in the first stage using the enter method. FT level (*r *= 0.40, *P *< 0.001) and sex (*r *= 0.22, *P *< 0.05) were tested for entry in the second stage using the stepwise method. The sex and FT level interaction (sex/FT interaction) was also tested for inclusion in the third stage using the stepwise method. The second stage retained FT levels (F change = 22.78, *P *< 0.001, ΔR^2 ^= 0.16), whereas sex and the sex/FT interaction were excluded from the final model (Figure 2).

Within-sex analyses showed that for girls, no variables met the criteria for entry into the hierarchical regression analyses except for FT level (*r *= 0.31, *P *< 0.05). This was entered in the first stage using the stepwise method. FT level was retained and produced a significant F change (F change = 6.59, *P *< 0.05, ΔR^2 ^= 0.10). For boys, gestational age (*r *= -0.25, *P *< 0.20) and child age (*r *= -0.19, *P *< 0.20) were entered in the first stage of the regression analysis. Inclusion of FT level in the second stage also produced a significant F change (F change = 9.60, *P *< 0.01, ΔR^2 ^= 0.14) (see Table 2).

**Table 2:** Final regression model for quantitative checklist for autism in toddlers (Q-CHAT) scores

Outcome	Predictors	Final regression model					
		
		**R**^**2**^	**Δ R**^**2**^	B	SE	β	*P*
Group							
Q-CHAT Score	Gestational age	0.02	0.02	0.47	0.32	0.12	> 0.05
	FT level	0.18	0.16	7.32	1.53	0.4	< 0.001
Girls only							
Q-CHAT Score	FT level	0.1	0.1	8.97	3.5	0.31	< 0.05
Boys only							
Q-CHAT Score	Gestational age	0.07	0.07	0.75	0.43	0.21	> 0.05
	Child age			0.1	0.57	0.02	> 0.05
	FT level	0.21	0.14	7.36	2.38	0.37	< 0.01

## Discussion

This study examined the relationships between amniotic measurements of FT and autistic traits measured using the Q-CHAT. Sex differences were observed, with boys scoring higher on the Q-CHAT than girls. These early sex differences are consistent with other studies in young children that have found a female advantage in eye contact [26], vocabulary development [25] and preference for looking at faces [37,38]. They are also consistent with sex differences on measures of autistic traits, both on the Childhood Autism Spectrum Test [39], the Autism Spectrum Quotient (AQ)-Child version [40], the AQ-Adolescent version [41] and on the English version of the adult AQ [1]. This pattern has also been observed cross-culturally using translated versions of the AQ [42-45].

FT levels were found to be approximately 2.5 times higher in boys than girls, consistent with previous studies using measurements of testosterone levels in amniotic fluid [22,23,27,46-48]. FT level was the only variable that was significantly related to Q-CHAT scores, both when the sexes were combined and when girls and boys were examined separately. Sex and the sex/FT interaction were not retained in the final regression model, suggesting that this is an effect of FT, rather than sex. No significant association between FT levels and FE levels was found, nor was FE correlated with autistic traits, and nor was there any association between child sex and FE levels (*r *= -0.02, *P *> 0.05), providing evidence for a link between autistic traits and FT levels rather than FE levels. This replicates previous studies showing no link between FE levels and behaviour [24-26,49,50].

The current study supports the idea that FT levels are associated with autistic traits in toddlers. Like previous research in older children showing a positive relationship with FT levels and autistic traits in 6 to 10-year-olds [27], these results at 18 to 24 months of age suggest that FT levels are associated with aspects of early development measured by the Q-CHAT, including sociability, early play behaviour and joint attention. These findings across different ages in development also suggest that the association between FT and autistic traits is robust. However, owing to the correlational design of this study, these results cannot be used to infer that FT is one of the causes of autism. Causality can only be demonstrated by manipulation of FT levels, which in humans would be wholly unethical. In addition, these studies only provide evidence for the role of FT in the development of autistic traits in typically developing children. This association remains to be tested in clinical samples, and a large-scale collaboration is currently underway to obtain a sufficient sample size to compare FT levels in cases of ASC versus controls.

The majority of total circulating testosterone is bound to sex hormone-binding globulin, and to albumin- and cortisol-binding globulin, and only the remaining 1-2% (the free fraction) is active [51]. In this study, the assay used can only measure total extractable testosterone and cannot be adapted to obtain measurements of free fraction testosterone levels. Whitehouse *et al*. (in press) obtained measurements of both the total testosterone and the free fraction testosterone concentrations using UC blood samples, and investigated relationships to pragmatic language ability in girls [19]. Although the only significant relationship was observed between the free fraction testosterone and pragmatic language ability, a significant correlation was also observed between total testosterone and the free fraction testosterone concentrations (*r *= 0.64, *P *< 0.01). This suggests that the measurements of total testosterone obtained in this study are still useful.

It is also known that hormones fluctuate during the day and between days, even in foetuses [52,53]. It is not possible to obtain repeated samples of FT because amniocentesis itself carries a risk of causing miscarriage (of about 1%) [54,55] so obtaining amniotic FT measures are opportunistic, when the procedure is being carried out for clinical reasons, with never more than a single measurement of FT at one time-point. The representativeness of a single sample of FT thus remains unclear, but would be difficult to explore in an ethical manner. Given the reported time course of testosterone secretion [56], the most promising time to measure FT is probably at prenatal weeks 8 to 24 [9,21,57], but this is still a relatively wide range. The inferences we can therefore draw about the single measurement of FT are necessarily limited.

This sample of children whose mothers had undergone amniocentesis is also not representative of the wider population (for example, maternal age is usually higher in such samples). In this study, no relationship between maternal age and Q-CHAT scores were observed, suggesting that the association found here may apply to all pregnant women. For the reasons mentioned above, it would be unethical to test this association in a randomly selected sample of the child-bearing population [9].

## Conclusions

We conclude that autistic traits are already sexually dimorphic as young as 18 months of age, and that FT is a significant predictor of autistic traits. The current findings are consistent with results in older (6 to 10 year-old) children, which have also shown that FT levels are a significant predictor of autistic traits. These results point to the importance of looking at factors that are involved with FT levels (such as genetic polymorphisms [15]) to understand the ultimate causes of individual variation in number of autistic traits.

## Competing interests

The authors declare that they have no competing interests.

## Authors' contributions

BA conceived of and carried out the study, and drafted the manuscript. KT carried out the radioimmunoassays. GH participated in the recruitment of women who had an amniocentesis. SBC contributed to the study design, obtained funding for and coordinated the study, and helped to draft the manuscript. All authors read and approved the final manuscript.
